# 

**Published:** 1906-07

**Authors:** 


### BOOKS RECEIVED.

Golden Rules of Surgery. Aphorisms, Observations and Reflections on the Science and Art of Surgery. Being a Guide for Surgeons and those who would be Surgeons. By Augustus Charles Bernays, A.M., M.D. Heidelberg, M.R.C.S. England. 8 vo., pp. 232. St. Louis: The C. V. Mosby Medical Book Co. 1906. (Price, $2.50).

Introduction to Materia Medica and Pharmacology, including the Elements of Medical Pharmacy, Prescription Writing, Medical Latin, Toxicology and Methods of Local Treatment. By Oliver T. Osborne, A.M., M.D., Professor of Materia Medica, Therapeutics and Clinical Medicine in Yale University. 12mo., 167 pp. Lea Brothers Co., Philadelphia and New York. 1906. ($1.00).

The Health-Care of the Baby. A Handbook for Mothers and Nurses, by Louis Fischer, M.D., 12mo., 166 pages. New York and London: Funk & Wagnalls Co. 1906. (Price: 75 cents, net.; by mail, 82 cents).

The Rose Croix. A Novel. By David Tod Gilliam, M.D. Illustrated by Ted Ireland. The Saalfield Publishing Co. 1906.

Thirty-second annual report of the State Board of Health of Michigan, for the fiscal year ending June 30, 1904. Henry B. Baker, M.D., Secretary.

Walter Reed and Yellow Fever. By Howard A. Kelly, M.D., Professor of Gynecological Surgery in Johns Hopkins University. 12 mo, pp. 293. Illustrated. New York: McClure, Phillips & Co. 1906.

Second Annual Report of the Henry Phipps Institute for the Study, Treatment and Prevention of Tuberculosis. February 1, 1904, to February 1, 1905. Lawrence F. Flick, M.D., Medical Director.

The Practical Medicine Series of Year Books. Ten volumes. Issued under the general editorial charge of Gustavus P. Head, M.D., Professor of Laryngology and Rhinology in the Chicago Post-Graduate Medical School. Vol 3. The Eye, Ear, Nose and Throat. Edited by Casey A. Wood, A. H. Andrews and G. P. Head. (Price: $1.50; entire series, $10.00). Chicago: The Year Book Publishers. 1906.

### LITERARY NOTES.

### INDEX TO VOL. LXI.

### AUGUST, 1905—JULY, 1906.

PAGE

ABDOMINAL pain and digestive disorders ....................448
Address, President Roosevelt's to Associated Physicians .......... 40
Age, Old and Senility, Delay of.. 1
Air tract, diseases of upper......551
American Medical Association, Dr. McMurtry's estimate of.... 44
Andrews, Champe S., Methods and Dangers of medical quacks....152
Anesthesia, local ................ 39
question of general or local ...........404
Ani, pruritis ....................164
Ankle, sprained ..................545
Ano-rectal disease, treatment of..109
Aortic stenosis ..................601
Appendicitis with erythema exudativum multiforme ..........102
Appendix and boric acid.........668
mucoid degeneration of.357
Arhovin, uses of ................610
Asthma, Prognosis of .......... 16

BABIES, novel method of saving ..........................107
Banning, John W., Arrest of....124
Beals, Lynn S., Laryngeal diptheria in nursing baby ..........361
Birth palsy, brachial ............366
Bissell, Elias L., Memorial.......301
Bladder, irritability of .......... 73
shape of the urinary....241
skiagraphy of ..........317
Blindness, Method of Abolishing unnecessary .........649
unnecessary ..........551
Boldt, H. J., Cesarean section on child of twelve ..............539
Blood, Diagnostic value of examintion of .........................285

**Books and Authors :**

Abbott: Principles of bacteriology ......................565
Abrams: Man and his poisons 685

PAGE

Anders: Practice of Medicine 464
Ashton: Textbook of Gynecology .........................385
Berg: Surgical diagnosis ....564
Blakiston: Physician's Visiting List ..................394
Boston: Clinical diagnosis ..567
Bouchard: Autointoxication in disease ....................628
Bradford: Orthopedic Surgery .......................326
Brickner: Surgical Assistant.325
Bryant: Operative Surgery .. 64
Busch: Physiology ..........560
Butler: Diagnostics of Internal Medicine ...........393
Cabot: Clinical Medicine—Diseases of Metabolism ..517
Cabot: Physical Diagnosis ...389
Caille: Differential diagnosis and treatment ............627
Church-Peterson: Nervous and mental diseases ...........681
Cohen: Physiologic Therapeutics, Vol. XI ..........136
Coleman: Syllabus of materia medica .....................743
Culbreth: Materia medica and pharmacology ............684
Cunningham: Textbook of anatomy ....................564
Cushny: Pharmacology and therapeutics ...............684
DaCosta: Gray's anatomy ....516
Debierre: Genital Organs of Women ...................137
Douglass: Nasal sinus surgery.744
Draper: Legal Medicine .... 66
Ellis: Sexual Selection in Man 69
Findley: Diagnosis in Diseases of Women ...............386
Fowler: Treatise on Surgery, Vol. 1 ....................740
French: Practice of Medicine.382
Garrigues: Gynecology ......134
Gould: Biographic Clinics, Vol. 3 .....................625

PAGE
Green: Textbook of Pathology ....................388
Griffiths: Medical Philology..202
Hall: Textbook of Physiology383
Hare: Practice of Medicine ..129
Hare: Progressive Medicine, Seventh Series, No. 2, 69; No. 3, 386; No. 4, 463; Eighth Series, No. 1,..............685
Hare: Practical therapeutics..563
Harrington: Practical Hygiene387
Head: Practical Medicine Yearbooks, 1905, Vol. 1, General Medicine, Billings-Salisbury, 137; Vol. 2, General Surgery, Murphy ....................137
Hensel: Urine and Feces......134
Hirst: Diseases of Women ..563
Holland: Medical Chemistry, etc. ........................132
Holt: Diseases of infancy and childhood ...................743
Howell: Physiology .........562
Hutchinson: Food and dietetics ......................742
Jackson: Diseases of Skin ...205
Jellett: Manual of Midwifery.200
Jennings: Color Vision and Blindness ................393
Kiliani: Surgical Diagnosis . 131
Klopstock-Kowarsky: Clinical Chemistry ................393
Lea: National Dispensatory.390
Lea: Practitioners' Visiting List ......................395
LeFevre: Physical diagnosis.565
Lippincott: Handbook of Nursing .................203
Lippincott: International Clinics, Fifteenth Series, Vol. 1, 66; Vol. 2, 385; Vol. 3, 463; Vol. 4,.............515
Sixteenth Series, Vol. 1.....741
Lydston: Diseases of Society. 56
Massey: Gynecology and Electrotherapeutics ............205
May: Diseases of Eye ......394
McGrath: Operative Surgery..518
McMurrich: Human Embryology ......................202
Medical Directory of N. Y., N. J., and Conn., 1905-06..682
Mettler: Diseases of the Nervous System .......... 63
Mills: Tumors of the Cerebellum ....................199
Moynihan: Abdominal Operations .....................389
Moynihan: Surgical treatment of gallstones ..............627
Mracek: Diseases of skin ....567

PAGE
v. Noorden: Part 6, Drink restriction in obesity, 204; Part 7, Diabetes Mellitus, ......567
Nothnagel: Encyclopedia of Medicine, Vol. IX, 68; Vol. X, 203; Vol. XI,...........384
Osler: Practice of Medicine ..394
Paddock: Maternitas ........204
Park: Pathogenic microorganisms ......................562
Paton: Psychiatry ..........391
Pedersen: Diseases of Eye and Ear (Alling-Griffin)....201
Pedersen: Diagnosis and Urinalysis (Arneill) 138
Pedersen: Practice of Medicine (Dayton) ............566
Pedersen: Medical Diagnosis, (Hollis) ..................135
Pharmacopeia, U. S., Eighth Revision .................. 67
Powell: Medical Formulary..326
Prentice: Eye, mind, energy and matter ................513
Rachford: Neurotic disorders of childhood ..............566

**Reports:**

Commissioner of Education, 1903 ......................206
Johns Hopkins Hospital, Vol. 12, 1904 ..................136
State Board of Health, N. H. 207

Rockwell: Dissecting manual 519
Ruhrah: Diseases of Infants..516
Sahli: Diagnostic Methods of
Savage: Ophthalmic Neuromyology .................... 388
examination ..............517
Schultze-Stewart: Applied Anatomy ................387
Scudder: Treatment of Fractures .....................325
Simon: Manual of Chemistry..565
Snow: Mechanical Vibration . 69
Stelwagon: Diseases of skin.561
Stevens: Materia Medica and therapeutics ..............682
Stevens: Practice of Medicine566
Stimson: Fractures and Dislocations ............ ...... 383
Stoney: Surgical technic for nurses .................... 68
Tanner: Memoranda on poisons .....................744
Taylor: Sexual disorders ....514

PAGE

**Transactions:**

American Dermatological Association, 1904 ............205
Medical Association of Alabama, 1904 ................ 68
Medical Society State of New York, 1905 ................464
Medico-Psychological Association, 1904 ..................515
Southern Surgical and Gynecological Association, 1904 ...519

Tanner: Memoranda on poisons ........................744
Tyson: Practice of medicine..683
Wainwright: Acute poisoning 518
Welch: Acute Contagious Diseases ...................130
Wharton: Minor Surgery and bandaging ................626
Wilcox: Materia Medica. etc. 519
Wilcox: Pharmacology and therapeutics ................681
Williams: Bacteriology ......563
Williams: Food and diet.....683
Williams: Practice of Medicine. .....................206
Wilson: Modern Clinical Medicine ...................201
Wood: Chemical and Microscopical Diagnosis ........133
Wood: Medical Record Visiting List ...................395
Wood: Therapeutics ........ 327
Wright: Textbook of Obstetrics 739
Young: Handbook of Anatomy387

Books Received ................... ............. 70, 138, 207, 276, 328, 332, 395, 465, 520, 568, 628, 686, 714
Breuer, Max C., Pubiotomy......348
Burke, Joseph, Posterior gastroenterostomy ..................529
Butler, Edward H., Contribution to University extension fund..508

CALOX, the oxygen dentifrice ........................124, 508
Catarrh, Internal medication in
Vesical ..................315
nasal, and headache ....243
Cesarean section on child of twelve ..................539
Section in the management of Placenta previa. 31
Chase, Walter B., The passing of a surgeon .....................502
Childhood, constipation in ......533

PAGE

Cholecystitis ....................409
Civil service examination, U. S.,..460

**College and Hospital Notes:**

Alumni Association, University of Buffalo ........513,679
Buffalo Emergency Hospital.382
Buffalo Fresh Air Mission Hospital .......................738
Buffalo General Hospital Training School for Nurses ......738
Buffalo Homeopathic Hospital Training School for Nurses 738
Buffalo Hospital Sisters of Charity Internes ...........738
Buffalo Municipal Hospital ..678
Buffalo Sisters of Charity Hospital ..................382
German Hospital Gift Fair ..272
Hospital Aid Association, Lockport .................625
Hospital Appointments ...... 56
Indiana Medical College ....199
Lexington Heights Hospital, Buffalo ...................680
Medical College of Va. .....680
N. Y. Post-Graduate College and Hospital .............381
N. Y. State Hospital for Crippled and Deformed Children 624
N. Y. Skin and Cancer Hospital ...................513
Oak Grove Hospital, Flint, Mich., ...................625
Pocahontas Hospital, Jamestown Exhibition ..........678
Riverside Accident Hospital, Buffalo .....................273
Riverside Hospital, Buffalo..560
St. Leo's Hospital, Greensboro, N. C.,. ...................625
St. Louis University ........680
St. Luke's Hospital, Utica ..274
St. Vincent's Hospital, N. Y..273
Syracuse University College of Medicine ..............625
University of Buffalo, Medical Department ......56, 199, 624
University of Buffalo, Orthopedic service .............512

Colonic flushing in diarrhea......106
Conjunctivitis, peculiar form of traumatic .....................317
Cook, Charles N., Clinical notes on wound dressing ...........359
Collargolum in Cerebrospinal meningitis .............. 37
in gonorrhea ....... 38

PAGE

**Correspondence:**

Diseases of Society—G. Frank Lydston ....................187
Exchange of Serum—H. K. Mulford Co. ..............249
Physicians publishing company—Roswell Park ........612
Tongaline and Yellow Fever —Mellier Drug Co. ........189
University of Buffalo Extension—Charles P. Norton and others ....................188

Coss, E. M., Tetanus............341
Cott, George F., A series of tracheotomies ..................640
George F., Student and young doctor ............209
Coughs, treatment of ......352, 548
Cowell, Walter A., Diagnostic value examination of the blood..285
Crile, George W., New operation for hernia of the pelvic floor.. 81
Crothers, Dr. T. D., Oration Norman Kerr memorial lectureship 123
Douglas, Richard, Cesarean Section in placenta previa ........ 31
Dowd, J. Henry, Irritability of the bladder ............... 73

DARLINGTON, THOMAS, The hurried life .................. 87
Diabetic gangrene, double amputation for ................647
Diabetes Mellitus, pathology and therapy of ................250
Diarrhea in Infants..........104, 105
treatment of in children 297
Diphtheria, after effects on heart.297
antitoxin in .........497
laryngeal in baby ....361
Doctor, the state and ...........481
the Twentieth Century..181
Dowd, J. Henry, Non-surgical treatment of prostate .....477

EDGAR, J. CLIFTON, Prevention of Ophthalmia in Newborn 170
Education Department to Chancellor Reid .................... 42
Elephantiasis ................... 23
Empyroform, a new tar preparation ..........................110
Esophagotomy for removal of false false teeth .................... 94
Eucain, lactate, in nose and throat operations .....................497

PAGE

Exhibition, historical, medical in London .......................673
Eyes of defectives ..............117

FLAHERTY, F. H., Strangulated femoral hernia in an old person .......................531
Foe, Conquest of the silent......218
Ford, Willis E., Surgical accidents of childbirth ..................413
Fowler, George Ryerson, Making of a Surgeon .......235
George Ryerson, In Memoriam .................449
Fronczak, Francis E., Foreign body in the lung ..............480

GALLSTONE DISEASE, symptoms and signs of.....574
Gallstone disease ...............294
Gastritis, treatment of chronic catarrhal .......... ..........571
Gastrojejunostomy; a case with specimen .......... ..........467
Gastroenterostomy, posterior....529
Gonorrhea, hygienic and dietetic treatment of .......229
management of acute.664
Grosvenor Public Library, new medical quarters in............124
Gunshot wound of abdomen .....292

HANLEY, L. G., I, Appendicitis, etc., II, Fibroid of ovary, etc ........................102
Hanley, L. G., Report of four cases ..........................357
Harrington, Devillo W., Memorial300
Hauenstein, John, Memorial ......301
Hayd, Herman E., Double amputation for diabetic gangrene 647
Herman E., Evolution of trained nurse .............303
Herman E., I, Gunshot wound of abdomen; II, Gallstone disease ..............292
Hemophilia, case of ............362
Hernia of the pelvic floor new operation for .................... 81
Hernia, strangulated femoral in old person ....................531
Herniotomy: sac contents, the appendix ............ ..........359
Howard, William Lee, Masturbation cause of sexual perversion290
Humidity, indoor ................544
Hurd, Arthur W., Aims and Uses of a Medical Society..........141
Hurried life, the ................ 87
Hysteria, new stigma in .........365

PAGE

ILLUSTRATIONS:
Bissell, Elias L., Portrait ....302
Bryant, Joseph D., Portrait ..457
Crile, Hernia of the pelvic floor:
Fig. 1, Normal relations of pelvic contents ..... 82
Fig. 2, Relations of structures in pelvic hernia ...... ........ 82
Fig. 3, Hernia reduced, hysterectomy partially completed ......... 83
Fig. 4, Completed operation, sagittal section .... 85
Fig. 5, Anchoring pelvic floor to rectus fascia 86
Dunning, L. H., Portrait ....378
Fowler, George Ryerson Portrait ......................451
Hanley, Appendicitis, with complications:
Fig. 1, Fibroid of ovary, etc. ................103
Hanley, Mucoid degeneration of appendix ...............357
Harrington, Devillo W., Portrait ......................300
Jack, vicious circles of Asthma ...................... 19
Lewis, Ampoules for distribution of silver ..............653
Meisenbach, Nontubercular joint lesions:
Figs. 1 & 2, Atrophic arthritis 336, 338
Fig. 3, Hypertrophic arthritis ...............339
Fig. 4, Infectious arthritis .340
San Francisco Disaster, picture of ...................616
Suzuki, S. M., Surgeon-General Imperial Japanese navy ..256
Ullman, Pseudoleukemia, Elephantiasis, and Rachitis ............... 24
Fig. 1, Pseudoleukemia..... 24
Fig. 2, Elephantiasis ...... 28
Fig. 3, Rachitis ............ 30
Vander Veer A., Portrait,49, 618

Indigestion, infantile ............471
Insanity, diagnosis of in borderline cases ....................601

**Items:**

Antikamnia Calendar, 1906 ..400
Apollinaris water ......400, 523
Arlington Chemical Co, 275, 334, .......................524
Battle & Co., Intestinal parasites 746

PAGE
Bloodless Phlebotomist ..275, 524
Breitenbach, M. J. Co., Bacteriological Chart ..............746
Breitenbach, M. J. Co., Physician's Daily Memorandum .334
Calox, scientific tooth powder334
Collier, Wm. T., Medical books ....................630
Evenden (J. W.) Co.........523
Japanese post cards in battle 72
Kress & Owen Co., Resignation of ...................630
Lambert Pharmacal Co. ....523
Mellin's Food Co. ..........524
Moet & Chandon White Seal275
Morse International Agency.630
Mulford, H. K. Co., Antistreptococcic Serum ......275, 524
Pabst Extract Indian calendar334
Palisade Mfg. Co. ..........275
Platt, L. D., Harvard operating chair .................570
Warner, Wm. R. & Co., "Beatrice" ....................400

JACK, George N., Prognosis of Asthma ..................... 16
Japanese, care of wounded.......307
Japan, real triumph of ..........218
Joint lesions, nontubercular .....335
Jones, Allen A., Chronic catarrhal gastritis ......................571

KIDNEY DISEASE .......... 77
Koyle, Frank H., Pseudokousma ......................281

LEADING ARTICLES:
American Medical Association at Portland ....... 47
Surgical Association 48
American Association of Obstetricians and Gynecologists ......................251
American Journal of Urology 263
American Medical Association at Boston ................672
Amalgamation? ..............260
Association of Military Surgeons, U. S. ..............193
Centenary of Medical Society State of N. Y...............373
Centennial of a Medical Society ......................456
De Senectute ...............122
Fowler, George Ryerson ....449
Medical practice bill, a new..554
Medical Unity in New York..318

PAGE

N. Y. Medical Journal and Medical News ....262
Oration, Dr. Stockton's at Portland .... 50
Osteopathy redivivus ....505
Politics in New Zealand....120
Prostatectomy, Fuller's suprapubic ....260
Raybrook, the change at .... 49
San Francisco calamity, the..614
Statistics, juggling of ....258
Suzuki, Surgeon-General Imperial Japanese navy ....254
Theater as a Disease-Spreader ....121
University Day—1906 ....459
Vander Veer, Albert, Regent University S. N. Y....617
War and Peace ....191
Yellow fever at New Orleans.118

Leukemia, splenic ....504
Lewis, F. Park, Method of abolishing unnecessary blindness ....649
Libel, Apology for .... 51
LeBreton, Prescott, Traction, as applied to tubercular joint conditions ....277

**Literary Notes:**

Alkaloidal Clinic ....332
American Journal of Medical Sciences ....522
American Medicine ....569
Appleton & Co., Catalogue ...332
Appleton & Co., Differential Diagnosis, etc. ....332
Blakiston's Son & Co., Casper's Lehrbuch der Urologie ....569
Blakiston's Son & Co., Manual & Atlas of Orthopedic Surgery ....140
Blakiston's Son & Co., "The Illustrated" ....745
Bloodless Phlebotomist ....398
British Medical Journal ....521
Central States Medical Monitor ....333
Chapin, John B., Addresses at Dinner to ....331
Cleveland Press, Chicago, Ferguson on Hernia....746
Davis, F. A. Co., Motor Apparatus of Eyes ....140
Encyclopedia, new International, Dodd, Mead & Co..570

PAGE

Gould's Medical Dictionary..522
Illinois Medical Journal ....332
Journal le Plus Cher du Monde ....522
Journal of Physical Therapy.331
Kansas City Medical Record 569
Lane, John Co., Practitioner's Handbooks ....139
Land of the Midnight Sun, Trip to ....399
Lea Bros. & Co., Food in Health and Disease, Williams ....399
Lea Bros. & Co., National Standard Dispensatory .140, 331
Maritime Medical News....522
Mulford, H. K. Co., Change Sheet U. S. P....208
New U. S. Pharmacopeia....398
N. Y. State Journal of Medicine ....569
Pharmaceutical Era ....208
Quarterly Journal of Inebriety 629
Saunders, W. B. & Co., Catalogue ....332
Saunders, W. B. & Co., Fowler's Surgery ....399
State Board Journal ....331
Transvaal Medical Journal...332
Wood, Wm. & Co., Bryant's Surgery ....686
Wood, William and Co., Cunningham's Anatomy .... 72

Lung, foreign body in....480

MASTURBATION, cause of sexual perversion ....290
McGuire, Edgar R., International Society of Surgeons ..231
Francis W., Symptoms and signs of Gallstone disease ....574
McKee, E. S., Constipation in childhood ....533
McKelway, St. Clair, the state and the doctor ....481
Medical Society, Aims and Uses of 141
Societies, nature and method of ....401
Meisenbach, Roland O., Non-tubercular joint lesions ....335
Meningitis, treatment of cerebrospinal ....242
Menstruation, medical treatment of disordered ....175
Milk bottles, paper ....619
Miller, Aaron B., Gastrojejunostomy ....467

PAGE

**Miscellany:**

Battle & Co., Intestinal Parasites.........140, 208, 400, 524
Boeckel Sanitarium, Gowanda 629
Buffalo Department of Health, Widal outfits ..............399
Civil Service Examinations, State ......................
140, 208, 276, 333, 400, 533, 629
U. S. ...........276, 570
Epilepsy Prize ..............333
Hotels for Military Surgeons Association .................746
Warner triennial prize ......522
U. S. Civil Service examinations .......................746

Mooney, James J., Submucous resection of the nasal septum.... 13
Murray, Dwight H., Minor rectal lesions .........................525
Myophobia .....................364

NASAL SEPTUM, submucous Resection of ................ 13
Neuralgia, Surgical treatment of trigeminal ....................536
Nurses, university school for ....114
Nurse, evolution of Trained .....303
N. Y. Medical Journal, commemoration edition ..................375

OBITUARY:
Balcom, Lafayette ...........197
Bartholomew, Lorenzo S.....462
Bissell, Elias L. ............267
Carroll, George Gregory ....267
Chadwick, James Read ......198
Day, Henry L. ..............197
Didima, Henry Darwin ......267
Dobbins, Edward T..........462
Doremus, R. Ogden .........557
Dudley, A. Palmer .......... 53
Dunning Lehman Herbert...377
Ellwood, Henry S. ...........557
Failing, John F. ............379
Failing, John S. .............735
Francis, Walter R. ..........380
Freeman, Simon A. ..........462
Gerold, Lawrence J. ........510
Gilbert, August L. ..........510
Hancock, Joseph Warren....379
Hauenstein, John ...........268
Heath, Christopher ..........198
Hill, Clayton L...............323
Hovey, Bleecker L. .........676
LeGrand, John Clark .......622
Lemon, Benjamin H. ........379
McKenney, F. B. ...........557
Mikulicz-Radecki, Johannes.. 53
Mulherin, John F. ..........379
Nothnagel, Hermann ........ 53
Ouchterlony, John Arvid ....268
Parsons, James Russell ......321
Preiss, Frederick ............736
Raub, Jacob F. ...............735
Robinson, William ..........267
Rochester, Nathaniel ........510
Saunders, W. B. ............268
Shepard, Francis L. ..........736
Steinkopff, Edward ..........623
Stevens, Andrew B. .........623
Sutton, R. Stansbury ........676
Tanner, John H. ............623
Tingley, Henry A. .........322
Tozier, Frank L. ...........622
Trotter, James S. ...........268
Vincent, William H. ........ 53
Walsh, John J...............322
Warren, Albert E. ..........197
Wetmore, S. W. ..............736
Weigel, Louis A. .............736
Wheeler, Isaac G. ..........677
Wiggins, Dennis Buell ......267
Woods, Laird M. .............510

OCULAR INJURIES, treatment of ....................498
Oil, pure olive ..................555
Ophthalmia, prevention of in New-born ...........................170
Osteopathic bill failed to pass...674

PARK, ROSWELL, Status lymphaticus and ductless glands ....................... 21
Park, Roswell, Oration in surgery 420
Perineal injuries and Methods of repair .........................147
Peritonitis localised ............358

**Personal:**

Bartow, Bernard; Snow, Irving M.; McKelway, St. Clair; Mabon, William...........510
Bemus, William M.; Higgins, L. S., Jr.; Edmunds, H. M.; Martin, A. J.; Trowbridge, Grosvenor R.; Gifford, W. B. ..........................620
Brush, Edward N. .......... 51
Burnham, N. L.; Smith Eugene A.; Trick, Harry R.; Hauser, Charles D.; Brush, Edward N.; Helwig, Jacob E...676

PAGE

Clark, Gaylord P.; Wende, Grover W.; Ballantyne, John W. ....................... 377
Francis, Lee Masten; Howard, William Lee; Long, Eli H.. 555
Gram, Franklin C.; Goldspohn, Albert; Morris, Lewis, C.; Wells, Brooks H.; Meisenbach, Roland O.; Price, Joseph ............. 321
Hayd, H. E.; Pryor, John H.; Reed, Charles A. L.; Fort, R. E.; Beates, Henry Jr.; Squier, Herbert Northrup... 509
Hinkel, Frank Whitehill; Crego, Floyd S.; Briggs, Albert H.; Dunham, S. A.. 197
Holmes, J. B. S.; Potter, William Warren; Chase, Walter B.; Cott, George F.; O'Donnell, William J.; Macallum, A. B.; Goodhue, W. J.; Russell, N. G. .......... 675
Jelliffe, Smith Ely; Bosher, Lewis C.; Roby, Joseph... 322
Lapp, Henry; Burnham, Melville Page ................ 53
Lydston, G. Frank; Smith, Chauncey Pelton; Pryor, J. H.; Pillsbury, Charles W.; Ebberts, Harry H.; Putnam, James Wright; Cott, George F.; Greene, Dewitt C.; O'Malley, Mary .......... 52
McMurtry, L. S.; Reed Charles A. L.; Price, Joseph; Henderson, A. C.; Briggs, A. H.. 376
Munson, J. F.; Clinton, Marshall; Benedict, A. L.; Plummer, W. W. .......... 622
Park, Roswell; King, James E.; Howe, Lucien; Fronczak, Francis E.; v. Noorden, Karl H.; Gaylord. H. M.; MacDonald, Carlos F.; Pillsbury, Charles W. .......... 461
Pohlman, Augustus G......... 674
Pryor, John H.; Johnston, George Ben; Caul, Martha F.; McMurtry, L. S.; Reed, Charles A. L.; Morris, Robert T. ................ 556
Raub, John F.; Twohey, John J.; Renner, W. Scott; Smith, Edward T................. 126
Reynolds, Arthur R.; Carstens, J. H.; Mann, Matthew D.; Rosenwasser, M.; Saunders, William H.; Jones, Allen A.; Haggard, William D.; Johnston, George Ben. 265

PAGE

Senn, Nicholas; Murphy, John B.; Totman, D. W.; Benedict, A. L............ 266
Shurly, E. L.; Foster, Frank P.; McGuire, Edgar R.; Park, Roswell; Young, Hugh H.; Bryant, Joseph D.; O'Reilly, Charles........ 125
Suzuki, S., Rear Admiral.... 196
Vander Veer, Albert; Ely, James W. C.; Stockton, Charles G.; Lewis, F. Park; MacLeod, James A.; Mathews, Joseph M.; Crego, Floyd S.; Pilgrim, Charles W. ....................... 621

Pharmacist, reliability of........ 320
Pigmentation, arsenica .......... 20
Placenta previa, Cesarean section in ............................ 31
Pleura, inflammations of ........ 294
Pneumonia commission ......... 175
creosotal in .. ...... 670
therapeutics .......... 545
treated with Antipneumonic serum ........ 631
Poliomyelitis, acute anterior..... 366
Prescription writing, suggestions on ............................ 587
Price, Joseph, Perineal injuries and methods of repair .. 147
N. Gordon, Treatment of Coughs ................ 352
Printers' strike ................ 264

**Progress in Medical Science:**

Miscellaneous Topics, by R. F. Sheehan .................. 544
Nervous and Mental Diseases, by R. F. Sheehan .... 364, 493
Pediatrics, by Maud J. Frye. ...................... 297, 494
Physiological Chemistry, by John A. Miller,.. 239, 295, 368
Summer Diseases in Children. 104

Prostatic hypertrophy .......... 661
Prostate, non-surgical treatment of ............................ 477
Pruritis ani .................... 164
Pseudoleukemia ................ 23
Pseudokousma .................. 281
Pubiotomy .................... 348
Puerperal fever, prevention of... 111
Pulmonary tuberculosis, conservative examination in............ 553
Pyrenol ........................ 499

PAGE

QUACKS, medical, their methods and dangers................152

RACHITIS .................... 23
Reciprocity between New York and New Jersey ..............263
Rectal lesions, minor ............525
Rectum and colon, treatment of..190
Roosevelt, President, Address of... 40
Rheumatic conditions, latent ....550
Ricketts, B. M., Surgical treatment of trigeminal neuralgia ........536
Rochester, DeLancey, Cholecystitis ..........................409

SAN FRANCISCO PHYSICIANS, aid for ............674
Scurvy, eye symptoms of infantile ........................... 98
Seaman, Louis L., Real triumph of Japan ..........................218
Sexual diseases, act prohibiting advertisements for cure of ......117
Sheehan, R. F., Case of hemophilia ............................362
Sixty-first year of Buffalo Medical Journal ........................ 51
Sleeping sickness, microbe of .....46
Smith, J. Gordon, Care of wounded Japanese .......................307
Snell, Albert C., Relation of vision to health ......................580
Snow, Irving M., Eye symptoms of infantile scurvy ............ 98
State Medical Examiners, appointment of by regents ..........619

**Society Meetings:**

Alumni Association, Medical Department, U. of B.......463
American Academy of Medicine ..................198, 623
American Academy of Ophthalmology and Otolaryngology ................128, 272
American Association of Obstetricians and Gynecologists ...................54, 463
American Gastroenterological Association ...............512

PAGE

American Laryngological Association ......................737
American Medical Association 511
Association of Military Surgeons, U. S. ..............559
British Medical Association ..558
Buffalo Academy of Medicine, .........55, 198, 273, 324, 380 446, 462, 512, 558, 624, 677, 737
Buffalo Association, Columbia Medical Alumni .......325
Central Medical Association, Lancaster ...................737
Erie Co. Medical Association ......................272, 323
Esculapian Club ............324
Fifteenth International Medical Congress.....126, 270, 381
Gross Medical Club .........128
Homeopathic Medical Society State of N. Y.........198, 512
Homeopathic Society Western N. Y. ......................272
International Congress of Insurance Physicians ........381
Lake Erie Medical Society.. ..................... 55, 269, 380
Lake Keuka Medical Association ......................55, 737
Medical Societies, Counties of Allegany and Cattaraugus, (N. Y.) and McKean, (Pa.).737
Medical Societies, Counties of Cattaraugus (N. Y.) and Mc Kean (Pa.)................128
Medical Society County of Chautauqua ................323
Medical Association of Central N. Y. ...........128, 198, 272
Medical Society County of Erie ................323, 678
Medical Society, County of Steuben ...................678
Medical Society County of Wayne ...................128
Medical Society of Missouri Valley ...................381
Medical Society State of N. Y.270
National Association of U. S. Pension Examining Surgeons ...................55, 624
National Confederation, State Examining Boards ........623
N. Y. & N. E. Association of Railway Surgeons....199, 269
Ohio Society U. S. Pension Examining Surgeons .....128
Southern Surgical and Gynecological Association ....272, 324
Western Surgical and Gynecological Association ........272

Page

**Society Proceedings:**

Buffalo Academy of Medicine, Section on Medicine, 546, 600
Medical Association of Central New York .......591, 655, 719
Medical Society, County of Erie, ............298, 440, 609
Medical Society, County of Genesee ..................608
Medical Society, County of Niagara ..................608
Niagara Falls Academy of Medicine ..................548

Spooner, Henry G., Hygienic and dietetic treatment of Gonorrhea 229
Status lymphaticus and the ductless glands ..................... 21
Stockton, Charles G., Delay of old age and alleviation of Senility ................ 1
Charles G., Nature and method of medical societies ....................401
Student and young doctor ......209
Substitution, law against ........552
Surgeons, International Society of 231
Surgeon, the making of a ......235
the passing of a ......502
Surgery, Oration in ............420
Surgical accidents of childbirth..413

TABO-PARALYSIS ............367
Tartaro, Guiseppe, Pane's antipneumonic serum in pneumonia.631
Tetanus ..........................341
Thomas, Luther A., Suggestions on prescription writing ........587

**Topics of Public Interest:**

Advertisements, Cure of Sexual diseases, Act prohibiting 117
American Medical Association, Dr. McMurtry's estimate of 44
Army corps medical examinations ......................501

Page

Doctor, improved methods of Twentieth Century ......181
Nurses, University school for 114
Pneumonia Commission ....175
President Roosevelt's Address to physicians .............. 40
President Roosevelt gives medal to Captain Church 370
Sato, the sorrows of ........244
State Education Department to Chancellor Reid ...... 42
Yellow fever immunity ......112

Tracheotomies, a series of ......640
Traction, applied to tubercular joint conditions ...............277
Trifacial neuralgia, castor oil in..365
Tubercular bacilli, vitality of ....546
Tuberculosis, mesentery glands of children ...................242
Types, pranks of the ............509

ULLMAN, JULIUS, Pseudoleukemia, Elephantiasis and Rachitis ...................... 23
University extension plans ......264
Physicians contribute to...374
Urine, incontinence of ..........494

VANDUYN, E. S., General or local anesthesia .............404
Veronal, therapeutic notes on ....493
Vision, relation of to health......580

WATER supplies and filtration.613
Wilson, Nelson W., Kidney Disease ....................... 77
Wilcox, Dewitt G., Presidential address of ....................460
Wound dressing, clinical notes on 359
Wyeth, John A., Esophagotomy for removal of false teeth...... 94

YELLOW fever immunity ....112
Young, A. A., Infantile indigestion ........................471

